# Tumor-Specific Induction of the Intrinsic Apoptotic Pathway—A New Therapeutic Option for Advanced Prostate Cancer?

**DOI:** 10.3389/fonc.2019.00590

**Published:** 2019-07-02

**Authors:** Philipp Wolf

**Affiliations:** ^1^Department of Urology, Medical Center - University of Freiburg, Freiburg, Germany; ^2^Faculty of Medicine, University of Freiburg, Freiburg, Germany

**Keywords:** prostate cancer, apoptosis, Bcl-2 proteins, targeted therapy, tumor specificity

## Introduction

Prostate cancer (PC) remains the second-most frequently diagnosed cancer among men worldwide and the fifth leading cause of cancer deaths (1). Despite great efforts to optimize radiotherapy, androgen deprivation, and chemotherapy, no curative treatment exists for advanced PC to date. This is due to the fact that during tumor progression and treatment, many changes occur in signaling pathways that lead to therapy resistance and treatment failure (2). Therefore, the search has to concentrate on signaling pathways that can be influenced even in advanced stages.

## The Intrinsic Apoptotic Pathway in Prostate Cancer

Cells have the ability to commit suicide initiated by a finely tuned signal network after the influence of stimuli that require the cell to die. This is why we are talking about “programmed” cell death or apoptosis. In multicellular organisms apoptosis serves to eliminate surplus or damaged cells to preserve tissue and organ homeostasis (3).

Apoptosis can be triggered by two different pathways. The “extrinsic” apoptotic pathway is initiated through the stimulation of transmembrane death receptors. The “intrinsic” apoptotic pathway is initiated after internal cell damage and marked by the release of cytochrome C from mitochondria (3). Key regulators of the intrinsic apoptotic pathway are pro- and anti-apoptotic Bcl-2 (B-cell lymphoma 2) family proteins (4). In non-apoptotic cells the main anti-apoptotic members, Bcl-2, Bcl-xl, and Mcl-1, bind the pro-apoptotic effectors Bax and Bak. Upon induction of apoptosis, the pro-apoptotic activators (BID, BIM, and PUMA) and sensitizers (BAD and NOXA) are transcriptionally or post-transcriptionally activated and bind via their so called BH3 domain to the anti-apoptotic proteins to free Bax and Bak. Bax and Bak can oligomerize and form pores in the outer membrane of mitochondria. This event is termed mitochondrial outer membrane permeabilization (MOMP) and marks the point of no return in apoptosis. MOMP is followed by cytochrome c release from the mitochondria, activation of caspases, and finally cell death (3).

PC therapies like radiation, androgen deprivation, and chemotherapy aim to activate the intrinsic apoptotic pathway by causing cellular stress (5–7). This cellular stress generally leads to an activation of different signaling pathways and expression of activators to free Bax and Bak for the induction of cell death (8–11). However, two essential conditions must be met for the therapies to be effective. First, the signaling pathways that induce apoptosis must be intact; second, Bax and Bak must not be inhibited by an excess of anti-apoptotic proteins for successful release. In fact, a deregulation of signaling pathways can be observed in PC during tumor progression and therapy, so that the threshold for the induction of apoptosis cannot be reached (2, 12, 13). Moreover, an upregulation of Bcl-2, Bcl-xl, and Mcl-1 was associated with resistance to apoptosis, radiation, androgen deprivation, and chemotherapy (14–19). Immunohistological studies have shown that the anti-apoptotic proteins Bcl-xl and Mcl-1 are continuously present at a high percentage in PC cells (81–100%) independently of grade or metastasis (Table 1). The number of Bcl-2-positive cells varied from study to study between 24 and 70% (17, 20–26). Interestingly, the pro-apoptotic effectors Bax and Bak were also detected at high percentages in all tumors (77.5–100%) and mutations of the Bak and Bax genes were shown to be rare events in PC (17, 20, 26). This means that PC cells may be characterized by a high apoptosis resistance due to their high expression of anti-apoptotic Bcl-2 proteins, but that they are at the same time capable for an induction of apoptosis due to their ubiquitous expression of the pro-apoptotic effector proteins.

**Table 1:** Percentage of Bcl-2 protein positive cells in prostate cancer, as determined by immunohistology in different studies.

		**Gleason**			**Metastases**			
**Bcl-2 proteins**		**2–4**	**5–7**	**8–10**	**Bone**	**Lymph node**	**Total**	**BH3 mimetics (inhibition)**
Anti-apoptotic	Bcl-2	20–25	14–42	33–41	13–65	14–38	24–70	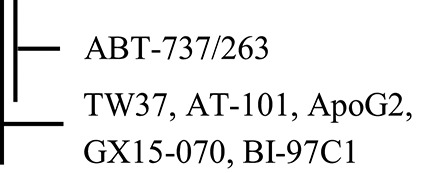
	Bcl-xl	100	100	100	100	100	100	
	Mcl-1	70	71	94	75	100	81	
Pro-apoptotic	Bax	100	100	100	100	100	95–100	
	Bak	nd	nd	nd	nd	nd	78–100	

*BH3 mimetics for inhibition of the anti-apoptotic proteins. nd, not determined*.

## Direct Activation of the Intrinsic Apoptotic Pathway in Prostate Cancer

The fact that the activation of the effectors Bax and Bak is prevented by Bcl-2, Bcl-xl, and Mcl-1 leads to an alternative therapeutic strategy to induce the programmed cell death by direct inhibition of the anti-apoptotic proteins. In principle, this can be done without previous cell damage and without consideration of upstream signaling pathway elements, which could be altered in advanced stages of the disease.

DNA-antisense and RNA interference methods were used to down-regulate the anti-apoptotic proteins in PC cells. In combination with chemotherapeutic agents or irradiation, synergistic cytotoxicity, and increased antitumor activity in *in vivo* tumor models were seen (27–30). However, in a phase II study combination of docetaxel with the Bcl-2 antisense oligonucleotide oblimersen did not enhance the clinical outcome of patients with castration-resistant PC (CRPC). Major toxic events were >grade 3 fatigue, mucositis, and thrombocytopenia and primary endpoints (PSA response > 30% and major toxicity event rate < 45%) were not reached (31).

A second focus for the direct activation of the intrinsic apoptotic pathway was on the development of Bcl-2 antagonists, such as BH3 mimetics. Similar to the Bcl-2 activator proteins, these small molecules can bind to the anti-apoptotic proteins Bcl-2, Bcl-xl, or Mcl-1 followed by a release of Bax and Bak (32).

First generation BH3 mimetics included small molecule inhibitors such as TW37, (-)-Gossypol (AT-101), Apogossypolone (ApoG2), BI-97C1 (Sabutoclax), or GX15-070 (Obatoclax Mesylate). They are known as pan-BH3 mimetics, because they can bind Bcl-2, Bcl-xl, and Mcl1. In preclinical experiments, they successfully induced apoptosis in PC cells (33–35). Due to the different structure of the Mcl-1 molecule compared to Bcl-2 and Bcl-xl, however, the pan-BH3 mimetics only show moderate affinity and low specificity against all these proteins and elicit apoptosis-independent off-target effects (36). AT-101 failed in a Phase I/II clinical trial, in which only two of 23 patients with PC experienced a decrease in PSA without objective responses. The main adverse side effect was gastrointestinal toxicity, which was dose-limiting (37). Moreover, combination therapy with AT-101 plus docetaxel/prednisone of patients with metastatic CRPC cancer did not extend overall survival (38).

Second generation BH3 mimetics were therefore created by structural-based design to bind with high affinity and specificity to individual members of the anti-apoptotic proteins (39, 40). The specific BH3 mimetic ABT-737 and its orally administrable analog ABT-263 (Navitoclax) can specifically inhibit Bcl-2 and Bcl-xl, but not Mcl-1. In preclinical studies, they were successfully combined with agents targeting Mcl-1 (e.g., chemotherapeutic agents or kinase inhibitors) to exceed the threshold for the induction of apoptosis in PC cells (35). A phase II study with Navitoclax and abiraterone acetate with or without hydroxychloroquine in patients with metastatic CRPC has been terminated, but outcome has not been published yet (ClinicalTrials.gov, Identifier: NCT01828476).

## Making the Induction of Apoptosis Tumor-Specific

Despite the recognition that a combination of specific BH3 mimetics with other drugs is necessary to achieve complete inhibition of the anti-apoptotic proteins Bcl-2, Bcl-xl, and Mcl-1 for the induction of apoptosis, these combinations have only hardly been used in clinical trials against PC to date. The main reason for this is that the combinations tested so far are not tumor-specific, but also can affect healthy cells. Second, most drugs, which showed synergistic effects with BH3 mimetics, target signaling pathways, e.g., androgen receptor (AR) or receptor tyrosine kinase (RTK) pathways, that are altered in advanced tumor stages (2, 13). It is therefore to be feared that the expected side effects of such combination therapies could outweigh the clinical benefits.

In recent years, new strategies were therefore developed to make the targeting of apoptosis more tumor-specific for enhanced efficacy and reduction of adverse side-effects. One such approach is the “BH3 profiling” of tumor cells, in which BH3 peptides interacting with Bcl-2, Bcl-xl, or Mcl-1 are used to identify the dependence of tumor cells on the respective anti-apoptotic Bcl-2 proteins. As a consequence, specific inhibitors could be selectively used to inhibit these proteins in the context of a personalized therapy. Specific Bcl-2 signatures for hematological tumors could be identified [reviewed in (41)]. It remains, however, questionable whether such profiling will also be successful in future for PC patients, because prostate tumors are known to be very heterogeneous and because it is difficult to isolate tumor cells for profiling in advanced stages.

A tumor specific therapy can generally be performed by using antibodies, peptides, or inhibitors against markers on the surface of tumor cells. With regard to a targeted induction of apoptosis, a direct coupling of Bcl-2 antagonists to tumor specific antibodies or a combination of Bcl-2 antagonists with immunotoxins represent promising, new therapeutic approaches. Berguig and colleagues generated an antibody-peptide drug conjugate consisting of an anti-CD22 antibody and a BIM peptide, targeting all anti-apoptotic proteins, to treat B cell lymphoma. The conjugate was applied via a multifunctional polymeric delivery system containing ethylene glycol segments to optimize safety and tumor biodistribution and butyl methacrylate (BMA) and diethylaminoethyl methacrylate (DEAEMA) for enhanced endosomal release. An inhibition of tumor growth marked by an increased apoptosis rate and an enhanced overall survival was reached with this construct in a B cell lymphoma mouse xenograft model (42). In another study combination of low dose EU-5346, a BH3 mimetic targeting Mcl-1, with trastuzumab induced significant cytotoxicity in Her-2 positive breast cancer cells (43).

In various tumor entities it was shown that the toxicity of *Pseudomonas* Exotoxin A (PEA) based immunotoxins can be significantly increased by the combination with the BH3 mimetic ABT-737/ABT-263 (44–46). PEA is a virulence factor of the bacterium *Pseudomonas aeruginosa* with ADP-ribosyltransferase activity. It ADP-ribosylates diphtamide, a modified histidine residue of the eukaryotic elongation factor 2 (eEF-2), in human ribosome complexes followed by inhibition of protein biosynthesis and induction of apoptosis (47). Initially, the inhibition of protein biosynthesis affects proteins with a short half-life due to rapid degradation, such as Mcl-1 (half-life < 1 h) (48). For the production of recombinant immunotoxins, the enzymatic domain of PEA is fused to a tumor-specific, internalizing antibody fragment and thus PEA is directed into tumor cells where it triggers apoptosis (49). In our group, we produced a PEA-based immunotoxin using an antibody fragment that specifically binds to the prostate specific membrane antigen (PSMA) on the surface of PC cells. With the anti-PSMA immunotoxin a downregulation of Mcl-1 was detected. When low doses of the immunotoxin and ABT-737 were combined, a synergistic cytotoxicity could be reached in PC cells, representing advanced androgen-dependent and independent stages (50). To our knowledge, this represents the first approach to make the induction of apoptosis specific for PC cells.

## Conclusions

The direct induction of the intrinsic apoptotic pathway is a promising new therapeutic option for advanced PC. In recent years, various drugs have been developed that can directly inhibit anti-apoptotic Bcl-2 proteins and induce apoptosis, independently of potentially altered upstream signaling pathways. Since these drugs could also affect healthy cells and lead to severe adverse side effects, future research must focus on strategies to make the induction of the intrinsic apoptotic pathway tumor-specific.

## Author Contributions

The author confirms being the sole contributor of this work and has approved it for publication.

### Conflict of Interest Statement

The author declares that the research was conducted in the absence of any commercial or financial relationships that could be construed as a potential conflict of interest.

## Abbreviations

AR: androgen receptor
BAD: Bcl-2 antagonist of cell death
Bak: Bcl-2 antagonist/killer
Bax: Bcl-2 associated X protein
Bcl-2: B-cell lymphoma 2
Bcl-xl: B-cell lymphoma extra-large
BID: BH3 interacting domain death agonist
BIM: Bcl-2 interacting mediator of cell death
BMA: butyl methacrylate
CRPC: castration-resistant PC
DEAEMA: diethylaminoethyl methacrylate
eEF-2: eukaryotic elongation factor 2
Her2: human epidermal growth factor receptor 2
Mcl-1: myeloid cell leukemia sequence
MOMP: mitochondrial outer membrane permeabilization
NOXA: phorbol-12-myristate-13-acetate-induced protein 1
PEA: *Pseudomonas* Exotoxin A
PC: prostate cancer
PSMA: prostate specific membrane antigen
PUMA: p53 upregulated modulator of apoptosis
RTK: receptor tyrosine kinase.
